# Bioreactor Co-Cultivation of High Lipid and Carotenoid Producing Yeast *Rhodotorula kratochvilovae* and Several Microalgae under Stress

**DOI:** 10.3390/microorganisms9061160

**Published:** 2021-05-28

**Authors:** Martin Szotkowski, Jiří Holub, Samuel Šimanský, Klára Hubačová, Pavlína Sikorová, Veronika Mariničová, Andrea Němcová, Ivana Márová

**Affiliations:** Faculty of Chemistry, Brno University of Technology, 612 00 Brno, Czech Republic; xcholubj@vutbr.cz (J.H.); xcsimansky@vutbr.cz (S.Š.); xchubacova@vutbr.cz (K.H.); xcsikorovap@vutbr.cz (P.S.); xcmarincova@fch.vut.cz (V.M.); andrea.nemcova@fch.vut.cz (A.N.); marova@fch.vut.cz (I.M.)

**Keywords:** co-cultivation, carotenogenic yeasts, carotenoids, *Desmodesmus* sp, microalgae, lipids, *Rhodotorula kratochvilovae*

## Abstract

The co-cultivation of red yeasts and microalgae works with the idea of the natural transport of gases. The microalgae produce oxygen, which stimulates yeast growth, while CO_2_ produced by yeast is beneficial for algae growth. Both microorganisms can then produce lipids. The present pilot study aimed to evaluate the ability of selected microalgae and carotenogenic yeast strains to grow and metabolize in co-culture. The effect of media composition on growth and metabolic activity of red yeast strains was assessed simultaneously with microalgae mixotrophy. Cultivation was transferred from small-scale co-cultivation in Erlenmeyer flasks to aerated bottles with different inoculation ratios and, finally, to a 3L bioreactor. Among red yeasts, the strain *R. kratochvilovae* CCY 20-2-26 was selected because of the highest biomass production on BBM medium. Glycerol is a more suitable carbon source in the BBM medium and urea was proposed as a compromise. From the tested microalgae, *Desmodesmus* sp. were found as the most suitable for co-cultivations with *R. kratochvilovae.* In all co-cultures, linear biomass growth was found (144 h), and the yield was in the range of 8.78–11.12 g/L of dry biomass. Lipids increased to a final value of 29.62–31.61%. The FA profile was quite stable with the UFA portion at about 80%. Around 1.98–2.49 mg/g CDW of carotenoids with torularhodine as the major pigment were produced, ubiquinone production reached 5.41–6.09 mg/g, and ergosterol yield was 6.69 mg/g. Chlorophyll production was very low at 2.11 mg/g. Pilot experiments have confirmed that carotenogenic yeasts and microalgae are capable of symbiotic co-existence with a positive impact om biomass growth and lipid metabolites yields.

## 1. Introduction 

Interest in the biotechnological processing of various materials (especially waste materials) is increasing within the circular economy’s current trend [1]. In comparison with the classical linear model, the circular economy’s main idea is recycling waste materials and their use for the production of new materials. This leads to increased protection of non-renewable resources, environmental protection, and economic development [2]. For the production of new products, it is more suitable to use renewable resources as input material (biomass, sugars, solar radiation, CO_2_), rather than non-renewable resources (coal, oil) [1]. Even in the case of biotechnological processing of these materials by heterotrophic microorganisms, only a part of the carbon is stored in the form of new carbon compounds. The rest is then released in the form of CO_2_ waste gas, which can be further used on site and transformed by autotrophic microorganisms instead of being released into the atmosphere.

Simultaneous cultivation of two types of microorganisms generates biological stress, which can lead to increased production of some stress-generated metabolites, e.g., antioxidants, osmotolerants, etc. Possible co-cultivation combinations can include following pairs of microorganisms: (i) Two different heterotrophs; (ii) two different autotrophs, and (iii) a heterotroph and an autotroph. The most promising is co-cultivation of heterotrophic and autotrophic organisms; nevertheless, some obstacles can occur, leading to compromises. To the most frequent belong differences in media pH and temperature, oxygen and nutrient demands, and mutual compatibility; in some cases, a slowly growing autotroph could be consumed by a heterotroph. Although setting suitable conditions can be problematic, intensive research of microorganisms in co-culture can lead to an almost “ideal combination” providing an innovative solution for lowering the carbon footprint, O_2_/CO_2_ exchange, and pH control. We can also hope for possible symbiotic exchange of molecules leading to a wide variety of known high-value products and also to new molecules. In biotechnology, the co-cultivation of heterotrophic yeasts and autotrophic microalgae together could be a very promising strategy [3]. 

Yeast, known as eukaryotic unicellular micro fungi, are widely present in nature [4,5]. They function as aerobic or facultatively anaerobic microorganisms. Certain yeasts, referred to as oleaginous, have the ability to accumulate up to 85% (*w*/*w*) lipids as a storage compound of the biomass [6]. These yeasts accumulate lipids primarily as storage lipids—triacylglycerols (TAGs). In yeast TAGs, saturated and monounsaturated fatty acids dominate, and some ω6-PUFAs are present. During recent years, oleaginous fungi have been extensively studied for the production of low- and high-value lipids from different agricultural and food rest materials [6,7]. They are able to utilize many hydrophobic substrates to co-produce other valuable chemicals (glucans, pigments, sterols, ubiquinone, and producing lipase and biosurfactants) in addition to lipids [8]. 

Some yeast species accumulate carotenoid pigments, such as β-carotene, torulene, and thorularodin, which cause their yellow, orange, and red colors and are therefore called red yeasts [9]. Carotenogenic yeasts are a diverse group of unrelated organisms (mostly *Basidiomycota*), and the majority of the known species are distributed in four taxonomic groups: The *Cystofilobasidiales* and *Tremellales* of the class *Hymenomycetesthe*, and the *Sporidiobolales* and *Erythrobasidium* clades of the class *Hymenomycetes* [10]. The most known representatives of red yeasts are *Phaffia rhodozyma* and its teleomorphic form *Xanthophyllomyces dendrorhous*. They have a high potential in biotechnology due to their specific profile of produced carotenoids, where the primary pigment is astaxanthin. Other basidiomycete yeasts can mainly produce β-carotene, torulene, torularhodin, and lycopene such as species belonging to the genera *Rhodotorula* and *Sporobolomyces* (including their teleomorphs *Rhodosporidium* and *Sporidiobolus*), which are characterized by high production of mixtures of different carotenoids. The composition and amount of the carotenoid pigments in numerous natural isolates were studied in detail [11]. Carotenoids are antioxidants that protect the cell membrane from the action of oxygen and peroxyl radicals. Their protective effect is determined mainly by their chemical structure, specifically the length of polyene chromophores, end groups, and various substituents [12,13].

Microalgae are either prokaryotic or eukaryotic photosynthetic microorganisms present in salt and fresh water. Photosynthesis of microalgae reaches about ten times higher efficiency than the one of terrestrial plants. They can obtain carbon dioxide, which is needed for this process, from the atmosphere, flue gases, or carbonates dissolved in the culture medium. Microalgae find a use, for example, in wastewater treatment, biofuel, and food and feed production [14]. They do not require specific environmental conditions for their growth. Some microalgae species are even able to survive in harsh conditions (extreme temperatures, acidic or alkaline pH, light, CO_2_ levels, metals) due to their multicellular structure and adaptation mechanism [15]. The enormous chemical diversity of their biomass is of great importance to the nutraceutical industry and pharmacy. Essential metabolites such as carotenoids, phycobiliproteins, and polyunsaturated fatty acids (PUFAs) can be obtained from microalgae biomass [16].

The co-cultivation of red yeasts and microalgae works with the idea of the natural transport of gases between specific microorganisms to ensure their essential vital functions. The microalgae produce oxygen, which stimulates yeast growth. Furthermore, the yeasts produce CO_2_, which is beneficial for algae growth. Both microorganisms can then produce lipids. In addition to the gas-exchanging effect, specific trace elements are usually released during cell lysis, which then yeast or microalgae can use for their own growth. Thus, it is supposed that the symbiotic effect of co-cultivation can achieve maximal biomass growth and high lipid yields compared to other methods of cultivation [3,17,18].

Thus, the aim of present study was to evaluate the ability of selected strains of microalgae and carotenogenic yeasts strains to grow and metabolize in co-culture. The effect of media composition (different C and N sources, C/N ratio) on growth and metabolic activity of selected red yeast strains was assessed. Simultaneously, microalgae adaptation to organic carbon in media and conditions of microalgal mixotrophy was tested. Cultivation was gradually transferred from small-scale laboratory co-cultivation in Erlenmeyer flasks to aerated bottles with different inoculation ratios and, finally, to a laboratory 3L bioreactor. 

It is worth noting that such complex simultaneous production of carotenoids, ubiquinone, ergosterol, and lipids under a combination of nutrition and biological stresses formed in a bioreactor with co-cultivation of red yeast and microalgae has never been evaluated before.

## 2. Materials and Methods

### 2.1. Yeast Strains

Based on our preliminary co-cultivation screening results, the most suitable yeast strain *Rhodotorula kratochvilovae* CCY 20-2-26 was used to study co-cultivation [19]. This strain was purchased from Culture Collection of Yeasts (CCY; Institute of Chemistry, Slovak Academy of Sciences, Bratislava, Slovak Republic), preserved in cryovials (YPD media with 50% glycerol solution) at −82 °C.

### 2.2. Microalgae Strains

For the experiment, several algae and cyanobacteria strains were enrolled in this study as follows: *Desmodesmus acutus* CCALA 439, *Desmodesmus quadricauda* CCALA 463, *Coccomyxa sp.* CCALA 912, *Chlorella sorokiniana* CCALA 260, *Desmodesmus dimorphus* CCALA 443*, Chlamydomonas reinhardtii* CCALA 928, *Scenedesmus* cf. *obliquus* CCALA 455, and Cyanobacteria *Synechococcus nidulans* CCALA 188. All strains were purchased from the Culture Collection of Autotrophic Organisms of the Institute of Botany of the Czech Academy of Sciences, Třeboň, Czech Republic. Strains were preserved on slant agar tubes and agar Petri dishes with Bolds Basal medium (BBM).

### 2.3. Media Preparation 

All strains of microorganisms used were cultivated on a standard BBM medium with various nitrogen sources (urea, ammonium sulphate, sodium nitrate). Glucose and glycerol were used as a source of organic carbon in the medium. The final composition per liter was: 0.250 g NaNO_3_, 0.075 g MgSO_4_.7H_2_O, 0.025 g CaCl_2_.2H_2_O, 0.006 g citric acid, 0.075 g K_2_HPO_4_, 0.175 g KH_2_PO_4_, 0.025 g NaCl, 0.050 g EDTA, 0.0049 g FeSO_4_, 0.0310 g KOH, 0.0088 g ZnSO_4_.7H_2_O, 0.0014 g MnCl_2_·4H_2_O, 0.0007 g MoO_3_, 0.0016 CuSO_4_.5H_2_O, 0.0005 g Co(NO_3_)_2_.6H_2_O, 30 g glucose. The concentration of other nitrogen and carbon sources in media was calculated to match the sodium nitrate and glucose concentration. Only one carbon and nitrogen source were used at a time. 

### 2.4. Co-Cultivation Experimental Scheme

The whole co-cultivation experiment was divided into several phases. Each of them addressed a specific issue or condition of possible co-cultivation of carotenogenic yeast and microalgae, namely:

1. Yeast growth curve and production on mineral BBM medium with different carbon and nitrogen sources in 4-day experiments.
2. Possible mixotrophy of selected strains of microalgae focusing on the utilization of glucose or glycerol in BBM media.
3. Small-scale co-cultivation experiments in Erlenmeyer flasks to determine the compatibility of yeasts and microalgae.
4. Co-cultivation in aerated round flasks under illumination to confirm small-scale experiments.
5. Co-cultivation under controlled conditions in a 3L bioreactor.

#### 2.4.1. Yeasts Cultivation on Mineral BBM Media (1st Phase)

Yeast cells were inoculated into a sterile YPD inoculation medium (50 mL) in a 250 mL Erlenmeyer flask for proper propagation, which took place on a reciprocal shaker for 24 h. Inoculation medium was inoculated with culture grown on solid YPD agar medium in a ratio of one inoculation loop to every 10 mL of liquid YPD medium. Inoculation of the liquid medium was done only with a 24-h-old culture. The next step was liquid-to-liquid media inoculation in a ratio of 1 part of the grown inoculation medium (25 mL) to 5 parts of sterile YPD inoculation medium (125 mL) in 500 mL Erlenmeyer flask. Cultivation took place in an Erlenmeyer flask on a reciprocal shaker with constant shaking for 24 h. After culturing the second inoculum, the culture was prepared for inoculation of production media. All cultivation experiments in Erlenmeyer flasks were performed at room temperature 22 °C on a reciprocal shaker with constant shaking at 120 rpm. 

Growth of *Rhodotorula kratochvilovae* was tested on Bolds Basal medium (BBM) with glucose or glycerol as carbon sources. Urea, sodium nitrate, and ammonium sulphate were further tested as nitrogen sources. Each tested nitrogen source was adjusted to be equivalent to the amount of nitrogen in sodium nitrate in BBM recipe. Combinations of carbon and nitrogen sources used in experiments are listed in Table 1. All experiments were further compared with two control media. The first was BBM with the addition of glucose and ammonium sulphate. The second was the mineral yeast medium, whose final components per liter was: Glucose** 30 g, glycerol** 29.97 g, (NH_4_)_2_SO_4_* 4.0 g, urea* 1.91 g, Yeast autolysate* 7.85 g, MgSO_4_·7H_2_O 0.696 g, KH_2_PO_4_ 4.0 g. Only one *N and **C source was used for cultivation at the same time.

Production media (40 mL) containing BBM combined with carbon and nitrogen sources were sterilized in 250 mL Erlenmeyer flasks. Production media were inoculated with the YPD inoculum in a ratio of 1:5; thus, each inoculated medium’s final volume was 48 mL. Yeasts were cultivated at room temperature with constant lighting and shaking on a reciprocal shaker for 4 and 10 days due to the microalgae’s lower growth rate. The produced biomass was centrifuged and washed two times with demi water. The washed biomass was then lyophilized and stored at −36 °C for further analyses.

#### 2.4.2. Microalgae Cultivation on BBM Media with an Organic Carbon Source (2nd Phase)

Microalgae strains were tested for their ability to utilize organic carbon sources or to grow autotrophically in the presence of organic carbon sources glucose and glycerol. The combination of C and N sources are shown in Table S1. In these experiments, the yeast autolysate was replaced by sodium nitrate due to autolysate’s lethal effects on microalgae cells. The medium containing no organic carbon and only sodium nitrate served as control.

These cultivations were performed in Erlenmeyer flasks with 40 mL of culture at room temperature and constant reciprocal shaking. Cultivation was performed in the dark, to confirm or disprove the mixotrophy in selected microalgae and cyanobacteria strains, by covering the Erlenmeyer flasks with aluminum foil. A stock culture of microalgae in standard BBM medium was used for inoculation. The absorbance of the culture at 680 nm was measured before inoculation. Subsequently, the production medium was inoculated with an amount of stock culture so that the initial A_680_ was 0.150.

#### 2.4.3. Co-Cultivation of Yeasts and Microalgae in Small Scale (3rd Phase)

The co-cultivation experiment took place based on previous phases results. Urea was chosen as the most suitable nitrogen source. The carbon source for co-cultivation was selected based on yeast cultivation results. The microalgae strain was selected based on the results of the second phase. 

The experiments were performed on a small scale in Erlenmeyer flasks on the reciprocal shaker. Microalgae were inoculated into the production BBM media containing a carbon source, and then yeast inoculum was added after 24 h. Cultures were inoculated so that the absorbance of the microalgae culture was A_680_ = 0.150, and the yeast was inoculated to obtain A_580_ = 0.100 (in the future, referred to as 1:1 ratio). The procedure for inoculating yeast and microalgae was the same as in the previous stages of the experiment. Cultivation lasted for ten days after inoculation of the yeast culture.

#### 2.4.4. Co-Cultivation in an Aerated 1 L round Pyrex Bottle (4th Phase)

Based on the evaluated data from the third phase of the experiment, experiments were designed on a larger scale. Selected yeast and algal strains were cultured in an aerated medium in 1 L round Pyrex bottle. In this experiment, yeast was cultured on BBM medium with the best carbon source, then a series of yeast and algae in different inoculation ratios of 1:1, 1:2, 1:4, where the ratios are absorbances of cultures when inoculated into the medium (A_680_ microalgae and A_530_ yeast). Next, pure microalgae bottles were cultivated in a standard BBM and the other BBM with an organic carbon source. In these bottles, the microalgae were inoculated to an absorbance of A_680_ = 0.100. The experimental scheme of the experiment is given in Table S2. Cultivation was again performed for ten days under constant illumination and aeration with sterile air at two L/min. The total volume of the medium was 400 mL. As in the previous case, the microalgae was inoculated 24 h earlier than the yeast.

#### 2.4.5. Large Scale Bioreactor Co-Cultivation (5th Phase)

The final phase was performed in a laboratory bioreactor with a volume of 3.0 L. Based on previous data, suitable species of microalgae were chosen to pair with *Rhodotorula kratochvilovae*, due to the high growth rate in the bioreactor.

BBM medium was poured into the reactor vessel, after which the bioreactor was equipped with an oxygen electrode and a pre-calibrated pH probe, aeration ring, stirrer, and temperature sensor. Aeration was provided through a 0.2 µm pore size filter. Subsequently, the fermenter and the equipment were sterilized in an autoclave and then cooled to room temperature. 

The bioreactor vessel was equipped with three bottles, which contained 5% KOH, 5% H_2_SO, and 80% glycerol solution, were sterilized together in an autoclave at 121 °C for 15 min. The glycerol solution served as a carbon source and contained a small amount of antifoaming agent. After cooling, the medium’s pH was adjusted to 6.5 and temperature to 22 °C with gentle stirring at 80 rpm. Under these conditions, the calibration of the oxygen probe was done. Zero pO_2_ value was set immediately after pH and temperature setting and 100% pO_2_ value was set after vigorous stirring and aeration. Microalgae were inoculated into the bioreactor, followed by a yeast inoculum. Cultivation was performed by the fed-batch technique, where the carbon source was gradually dosed to the medium in the first three days of cultivation according to the following procedure: 0 h—50%, 24 h—25%, and 48 h—25%. Cultivations always lasted six days, i.e., 144 h. Process methods during cultivation are listed in Table S3.

### 2.5. Analytical Methods

#### 2.5.1. Cell Dry Weight

Fermentation samples (10 mL) were centrifuged at 6000 rpm for 2 min. The supernatant was collected for further analyses (lipase, biosurfactants, pH) and stored at −20 °C. Then, the yeast cells were washed twice with the mixture of distilled water and hexane 1:1 (*v*/*v*) and suspended in 1 mL of distilled water. Then purified biomass was quantitatively transferred into Eppendorf tubes, frozen at −20 °C, and then freeze-dried. After determining their weight, to calculate CDW, the dried cells were used for analysis of carotenoids, ergosterol, ubiquinone, lipids, and glucans.

Fermentation samples (40 mL) were taken for gravimetric determination of biomass production and subsequent analysis. Each sample was centrifuged at 8000 rpm and washed twice with 20 mL of distilled water. Then the purified biomass was then quantitatively transferred into Eppendorf tubes, frozen at −80 °C, and then freeze-dried. After determining their weight, to calculate CDW, the dried cells were used for the analysis of carotenoids, ergosterol, ubiquinone, and lipids. Lyophilized samples were stored at −36 °C freezer for further analysis.

#### 2.5.2. Pigment Analysis

Total carotenoid, sterol, coenzyme Q, and chlorophyll content were determined using the HPLC/PDA method. Samples of freeze-dried combined yeast algae biomass were properly mixed and weighed (approximately 15–25 mg) and rehydrated with 1 mL of MiliQ water for 30 min. Excess water was removed by centrifugation at 12,000 rpm, and 1 mL of methanol and about 0.5 mL of glass beads (0.2–0.5 mm diameter) were added to the sample. The sample was vortexed for 20 min and then transferred to a 15 mL tube and washed with 2 mL of chloroform. The mixture was further vortexed for 10 min. Then, 1 mL of water was added, and the tube was allowed to stabilize for two phases after shaking. The lower chloroform phase was quantitatively transferred to a clean tube and dried under an inert nitrogen atmosphere. The dried sample was dissolved in 1 mL of 2:1 EtAc:ACN and filtered through a 0.45 μm PTFE filter into the vial. Samples were measured on Dionex Ultimate series HPLC with Vanquish DAD detector (Thermo Fischer Scientific, Waltham, MA, USA) on Kinetex C18-EVO column 150 mm × 4.6 mm × 5 µm (Phenomenex, Washington, DC, USA) using gradient separation with mobile phase A (ACN:MeOH:Tris HCl pH = 8; 84:2:14) and mobile phase B (MeOH:EtAc; 60:40) at a flowrate of 1.2 mL/min and 25 °C. The gradient program is listed in Table S4.

Carotenoid pigments were detected at 445 nm and chlorophyll at 445 nm and 455 nm. Chromatographic data were evaluated using Chromeleon 7.2. software. Total carotenoid, sterol, coenzyme, and chlorophyll production were identified and evaluated using commercial standards (Carotenature, Münsingen, Switzerland) and external calibration as in [8].

#### 2.5.3. Lipids and Fatty Acids

Total lipids and individual fatty acids were determined by optimized GC/FID analysis. Approximately 10–15 mg of freeze-dried combined yeast algae biomass was put into 2.0 mL crimp neck vial together with 1.8 mL 15% (*v*/*v*) H_2_SO_4_ in methanol, capped with an aluminum cap, and heated at 85 °C for 2 h. After the transesterification process, the mixture was transferred quantitatively into a 5 mL vial and neutralized with 0.5 mL of 0.005 M NaOH. The FAME was converted to the non-polar phase by adding 1 mL of n-hexane and shaking vigorously. The total lipids and fatty acids profile were determined by gas chromatography/flame ionization detection (GC/FID) analysis. GC analysis of FAMEs was carried out on a TRACETM 1300 Gas Chromatograph (Thermo Fischer Scientific, USA) equipped with a flame ionization detector, an Al 1310 autosampler, and a Zebron ZB-FAME column (30 m, 0.25 mm, 0.20 μm) (Phenomenex, USA). The temperature program is listed in Table S5. FAME was identified using commercial standard Supelco 37 Component FAME Mix (Sigma Aldrich, St. Louis, MO, USA, SRN). The internal standard method was used for quantification via the addition of heptadecanoic acid (Sigma Aldrich, SRN) into the transesterification mixture in concentration 0.5 mg/mL. Chromatography data were evaluated using Chromeleon software 7.2 [8].

### 2.6. Statistical Analysis

The growth experiments in Erlenmeyer flasks and in 1L Pyrex flasks were carried out in triplicate and duplicate, respectively. The presented results are the mean of the replicates, and the standard deviations are shown as error bars in the figures. Data handling and statistics were performed using the Excel software package (Microsoft Excel 2013, Microsoft Corp., Redmond, WA, USA).

## 3. Results 

### 3.1. Yeast Cultivation on BBM and Standard Media

#### 3.1.1. Biomass Production

**Figure 1:**
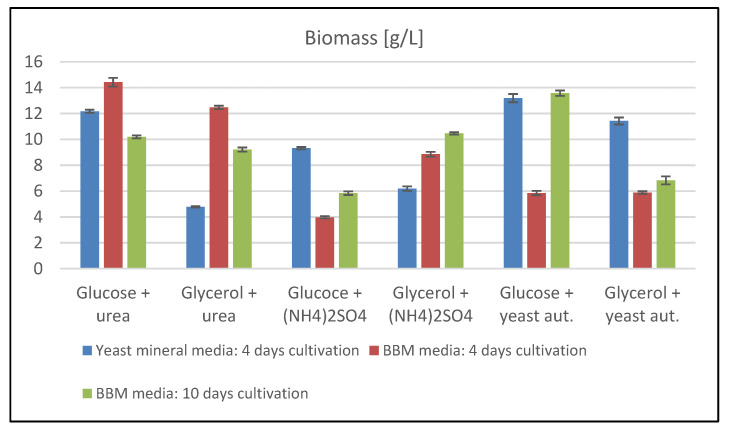
Biomass production of *Rhodotorula kratochvilovae* cultivated on different media types.

In this phase of the experiment, basic cultivations were performed to compare yeast growth in the simple mineral medium and BBM medium used for microalgae cultures. Furthermore, the influence of different combinations of carbon and nitrogen sources on yeast’s growth and production properties were tested here. At the beginning of the experiment, two sets of 4-day cultures were performed, followed by an experiment with 10-day cultures on BBM medium. The extended cultivation time was chosen in order to be able to compare the growth with the microalgae in the subsequent phase of the experiment. In Figure 1, the comparison of the biomass production in *R. kratochvilovae* cells can be found. The results show that there are fundamental differences in biomass production within one strain due to the different carbon and nitrogen source in all cultivations. 

The best biomass production results were obtained with glucose as a carbon source, where combinations with urea produced 12.17 ± 0.12 g/L and yeast autolysate 13.38 ± 0.32 g/L on Yeast mineral media. Similar results were obtained from the 10-day cultivation on BBM media, where production on these combinations reached 10.19 ± 0.12 g/L and 13.57 ± 0.21 g/L. In terms of glycerol utilization, the highest biomass production of 11.42 ± 0.27 was achieved in combination with yeast autolysate on yeast mineral media. In the case of cultures on BBM medium, higher biomass production was achieved compared to glucose media only in combination with ammonium sulphate in both 4-day (8.85 ± 0.18 g/L) and 10-day (10.46 ± 0.09 g/L) experiments. During the shorter 4-day cultivation of yeast on the BBM medium, the best growth was obtained on media with urea and glucose. 

Basic yeast cultivation on a BBM and yeast mineral medium showed significant differences in growth rate and biomass production. Overall, glucose proved to be a better carbon source than glycerol, except for cultivation with ammonium sulphate. Prolonged cultivation of yeast on BBM medium, aiming to simulate the length of cultivation for microalgae, revealed no substantial changes in the extended experiment. In the case of glucose media, data show a decrease in biomass production, and in the case of glycerol media, a positive effect in media containing ammonium sulphate or urea. Overall, the highest biomass production was achieved in media containing yeast autolysate as a nitrogen source, followed by urea containing media. The lower biomass production on ammonium sulphate media was caused by an accelerated decrease in the pH of the medium, where after treatment of ammonium cations by cells, sulphate anions are released into the medium, which subsequently lowers the pH of the medium and inhibits further cell growth.

#### 3.1.2. Carotenoid, Sterol and Ubiquinone Production

During experiments performed in the first phase (yeast cultivation), carotenoids, sterols, and ubiquinone were analyzed in yeast cells by HPLC (see Section 2.5.2). The highest carotenoid yield of 7.806 ± 0.097 mg/g yeast biomass was achieved on a medium with glycerol and ammonium sulphate. Together with beta-carotene, lycopene and torularhodin production were measured in all samples. In terms of ergosterol production, the production of 6.024 ± 0.098 mg/g of biomass was achieved on both media containing ammonium sulphate. Ubiquinone was identified in all samples, the production of which ranged from 1.0 to 2.0 mg with a maximum in the medium with glucose and yeast autolysate. Overall, the most successful medium was a combination of glycerol and ammonium sulphate. Table S6 shows the production during the cultivation of *R. kratochvilovae* on a standard mineral yeast medium.

The experiment continued with cultivation on the mineral BBM medium with a combination of carbon and nitrogen sources. The production data of the strain *R. kratochvilovae* (Table S6) show a decrease in the monitored metabolites production compared to the classical yeast medium. The results show that glycerol is a more suitable carbon source in the BBM medium for this strain. The highest production of the monitored metabolites was achieved in combination with urea, followed by the medium with ammonium sulphate and both media with yeast autolysate. Significant production of ergosterol 4.017 ± 0.103 mg/g biomass on the medium with glycerol and urea can be noticed compared to other media. Glycerol in the medium was again most suitable for the production of ubiquinone in yeast.

#### 3.1.3. Fatty Acid Profile and Lipid Production

As with HPLC analysis, samples of the biomass produced were subjected to GC-FID analysis to determine the fatty acid profile and lipid production according to Section 2.5.3. It was also studied whether different combinations of carbon and nitrogen sources affect the composition of fatty acids in biomass. 

Figure S1 shows the results of GC analysis of *R. kratochvilovae* cultivation. In the first part showing the results of cultivation on yeast medium, a relatively stable 15% lipid content in the biomass was measured, with the exception of the medium with glycerol and urea, where the maximum for this experiment was set at 19.94%. The highest SFA content was also measured in this medium, reaching 39%. The highest PUFA production of 34.61% was measured in the glycerol and yeast autolysate media. Long-term cultivation on BBM media harmed lipid production in all samples, and the total lipid content of all samples is below 10%. However, except for media with yeast autolysate, a significant increase in PUFA content compared to yeast mineral media was measured. In most cases, however, the increased PUFA production was at the expense of MUFA production, which is very low, and almost no MUFA was measured in a sample of BBM media with glycerol and yeast autolysate. 

### 3.2. Microalgae Cultivation on BBM Media

It was found that none of the selected strains of microalgae were capable of a mixotrophic lifestyle. From a continuous measurement of the growth curve at 680 nm, a linear decrease in absorbance after three days was observed for all strains, continuing until the culture’s end during the whole experiment/observation.

In the second series, the microalgae were cultured under light for ten days. In a long-term experiment, the culture growth was stopped or killed. Due to this, no analyses of lipid composition and pigment production were performed. Based on these results, the co-cultivation procedure was changed in the next phase of the experiment.

### 3.3. Small Scale Co-Cultivation of Yeasts and Microalgae

#### 3.3.1. Biomass Production during Small Scale Co-Cultivation

Based on previous experiments, the cultivation procedure was modified. The microalgae were inoculated first and then the yeast and carbon source. The cultivation was performed on reciprocal shakers in Erlenmeyer flasks according to Section 2.4.3. Co-cultivation continued with urea as a single source of nitrogen. 

**Figure 2:**
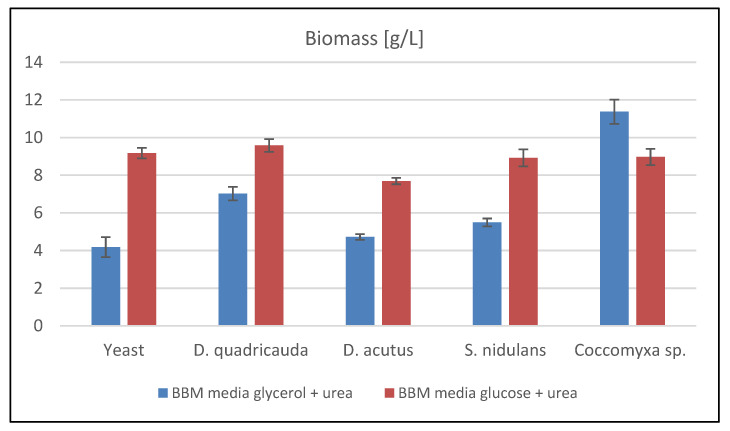
Biomass production of small-scale co-cultivation experiments [g/L]. Abbreviations: Yeast—*Rhodotorula kratochvilovae* control cultivation; *D. quadricauda*—Co-cultivation with *Desmodesmus quadricauda*; *D. acutus*—Co-cultivation with *Desmodesmus acutus*; *S. nidulans*—Co-cultivation with *Synechococcus nidulans*; *Coccomyxa* sp.—Co-cultivation with *Coccomyxa* sp.

A comparison of biomass production is given in Figure 2. In the case of *R. kratochvilovae*, co-cultivation with all tested microalgae strains was successful. The microalga *D. quadricauda* always achieved a greater biomass production during co-cultivation than pure yeast. In the experiment with glycerol in the medium, the biomass production was less than 3 g/L. Similarly, when cultivated with *Coccomyxa* sp. on glycerol, higher production of 7 g/L was achieved. In general, all micro-algal co-cultivations were successful, and glycerol was a more suitable carbon choice.

#### 3.3.2. Carotenoid, Chlorophyll, Sterol, and Ubiquinone Production in Co-Culture of *R. kratochvilovae*

Another essential evaluation factor was total production of pigments, sterols, and ubiquinone. The media had the same composition, based on BBM media with urea as a nitrogen source (see Section 2.4.3). The only difference was in the carbon source, where glucose or glycerol was used. As the most abundant major carotenoid pigments present in co-culture were lutein and β-carotene, total carotenoids, chlorophylls A and B, ergosterol, and ubiquinone were evaluated. Other identified carotenoid pigments were excluded from the table due to low production. These were violaxanthin, neoxanthin, torulene, torularhodin, astaxanthin, and lycopene, present in negligible concentrations. They are shown in the graph only in the case of higher productions. The co-cultivation part of the results compared the production of yeasts and algae both on their own and co-cultivations. 

The co-cultivation results of the *R. kratochvilovae* strain with selected microalgal strains is shown in the following table (Table S7). The data show that the largest biomass production was achieved in the co-cultivation of yeast with the microalgae *D. quadricauda*. Comparable production was further obtained in co-cultivation with microalgae *Coccomyxa sp.* and cyanobacteria *S. nidulans*. By comparing the chromatographic data with the control culture, increased ergosterol production was measured in all cultures except *Coccomyxa sp.* Carotenoid production increased in two cases in co-cultivations with *D. quadricauda* and *D. acutus*. Production of 2.191 ± 0.207 mg/g and 2.133 ± 0.182 mg/g of biomass was achieved, compared to 1.827 ± 0.065 mg/g in control culture. Increased ubiquinone production was observed in co-cultures with *D. acutus* and *S. nidulans*. In terms of the production of the metabolite produced by microalgae, we see deficient production of chlorophylls. The best experiment’s highest values were achieved with the microalga *D. acutus*, namely 140 ± 32 µg/g biomass. No chlorophyll was identified when cultivating with *D. quadricauda*. 

In the overall evaluation, the most suitable microalgal strains in these experiments were *Desmodesmus* and the *Coccomyxa* strain for both the yeast *Rhodotorula kratochvilovae*. Glucose was a better source of carbon. 

#### 3.3.3. Fatty Acid Profile and Lipid Production in Small Scale Co-Cultivation

The results of lipid analysis in mixed biomass are shown in the Figure S2. The data are sorted according to the mixture of carotenogenic yeast and the corresponding microalgae in co-cultivation medium. The main monitored parameters were the composition of individual groups of fatty acids, the percentage of lipids in biomass, and total lipid production. Figures S2 and S3 shows cultivation results with the *R kratochvilovae* strain on BBM media with glycerol and glucose. Overall, lower production of lipids in biomass was achieved in co-cultivations. Only in glucose media did co-cultivation with *D. quadricauda* and *S. nidulans* achieve approximately the same production as the pure control medium. The unifying parameter for both types of media is the low PUFA content, except for cultivation with *D. acutus* on glucose, which was always below 30%. 

### 3.4. Co-Cultivation of Yeasts and Microalgae in Aerated Flasks

#### 3.4.1. Growth of Co-Culture

Based on the data obtained from previous experiments, co-cultivations of selected pairs were performed in aerated 1L Pyrex flasks. In each culture, yeasts were cultivated separately on BBM media with carbon sources, microalgae on the BBM medium with and without carbon source, and then co-cultivated with yeasts in three inoculation ratios: Yeast: microalgae 1:1, 1:2, and 1:4 (see Section 2.4.4). In the experiments, the yeast was inoculated so that the initial absorbance of the yeast culture was A_580_ = 0.100. Microalgae was added to BBM medium with and/or without a carbon source to verify the influence of organic carbon source and potential effect of bacterial contamination on the microalgae if any. The effect of air aeration on the production of biomass and metabolites of yeasts and microalgae in co-cultures and separate cultures was studied. 

**Figure 3:**
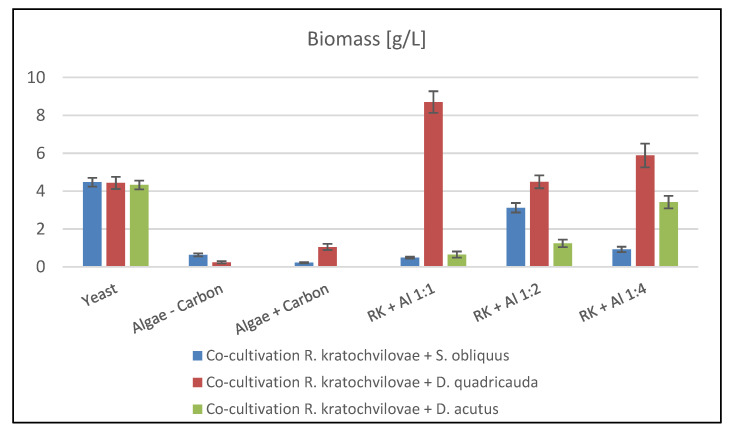
Biomass production of *R. kratochvilovae* during co-cultivation in aerated Figure 1. Co-cultivation of *Rhodotorula kratochvilovae* with microalgae with an inoculation ratio of 1:1; RK + AL 1:1—co-cultivation of *Rhodotorula kratochvilovae* with microalgae with an inoculation ratio of 1:2; RK + AL 1:1—co-cultivation of *Rhodotorula kratochvilovae* with microalgae with an inoculation ratio of 1:4.

Figure 3 summarizes the biomass production by co-cultivation of *R. kratochvilovae*. In the co-culture with *S. obliquus* and *D. acutus*, low biomass production was achieved. An exception is co-cultivation with *S. obliquus* in a ratio of 1:2, where 3.12 ± 0.61 g/L of dry biomass was produced. Furthermore, in co-cultivation with *D. acutus* in a ratio of 1:4, the final biomass production was 3.42 g/L. Compared to the pure yeast control culture, it is lower by one-quarter, but data from chromatographic analyses are essential. Contamination and death of the culture occurred during the sole cultivations of *D. acutus*. Overall, the most successful was co-cultivation with *D. quadricauda* microalgae (Figure 3). The maximum was the production of 7.21 ± 0.63 g/L in a ratio of 1:2, which exceeded the control culture by almost 3 g/L.

#### 3.4.2. Carotenoid, Chlorophyll, Sterols, and Ubiquinone Production in Cultures Grown in Pyrex Flasks

The major lipid soluble metabolites evaluated in co-cultures from aerated flasks were lutein and β-carotene, total carotenoids, chlorophylls A and B, ergosterol, and ubiquinone. Other identified carotenoid pigments were excluded from the plot due to low production (in the case of higher productions they are shown in Table 2.

It is already known from biomass production data that in the case of *R. kratochvilovae*, the best partners for co-cultivation are representatives of the genus *Desmodesmus.* Chromatographic analysis of the pigment and other lipid substances confirmed this assumption. The most successful co-cultivation was a 1:2 ratio, where carotenoid production was 11.221 ± 0.362 mg/g when dry biomass was measured. In general, all co-cultivations always had higher carotenoid production. However, the carotenoid profile is different. There is a reduction in the production of the primary carotenoid pigments β-carotene and torulene favoring other pigments. At the same time, a low production of chlorophylls (less than 1 mg/g biomass) was obtained compared to conventional microalgae cultivation. At the end of the cultivation, there was probably a reduction in chlorophylls production by algae and an accumulation of carotenoid pigments. Co-cultivation also positively affected ubiquinone production, where the highest values of 6.035 ± 0.213 mg/g biomass were reached at a co-cultivation ratio of 1:1. At the same co-cultivation ratio, the highest ergosterol production was also achieved.

Cultivation of the microalgae *Desmodesmus acutus*, even in repeated experiments, led to death in aerated bottles with pure algal culture (Figure 3). Interestingly, in the case of co-cultivation under given conditions, the microalgae survived, as evidenced by the measured data (Table 2) of chlorophyll A and B production.

Based on the data obtained from co-cultivation with representatives of the genus *Desmodesmus*, a test co-cultivation with microalgae *Scenedesmus obliquus* was performed, without previous experimental steps. Due to the morphological similarities between the genera *Desmodesmus* and *Scenedesmus*, a representative of the genus *Scenedesmus* was also tested in this experiment. The positive effect of co-cultivation with *Scenedesmus* on studied metabolites production was measured (Table 2). The control culture of pure microalgae has the highest production of monitored metabolites, but on the other hand, the production of biomass was very low, only 0.63 ± 0.12 g/L. In an overall comparison, the production of lipid substances was higher in co-cultivation experiments, in which the highest production was achieved at a culture ratio of 1:1. Here, carotenoids production was 7.458 ± 0.176 mg/g of biomass, of which more than 60% was β-carotene. The highest ubiquinone production was also achieved in the same medium, namely 4.733 ± 0.099 mg/g biomass. This smallest inoculation ratio was most suitable for the microalgae itself. Compared to the control of sole yeast culture, we see that the co-cultivation was more productive in all proportions. If we include biomass production, the largest profits were achieved at a ratio of 1:2.

#### 3.4.3. Fatty Acid Profile and Lipid Production in Co-Culture Grown in Pyrex Flasks

**Figure 4:**
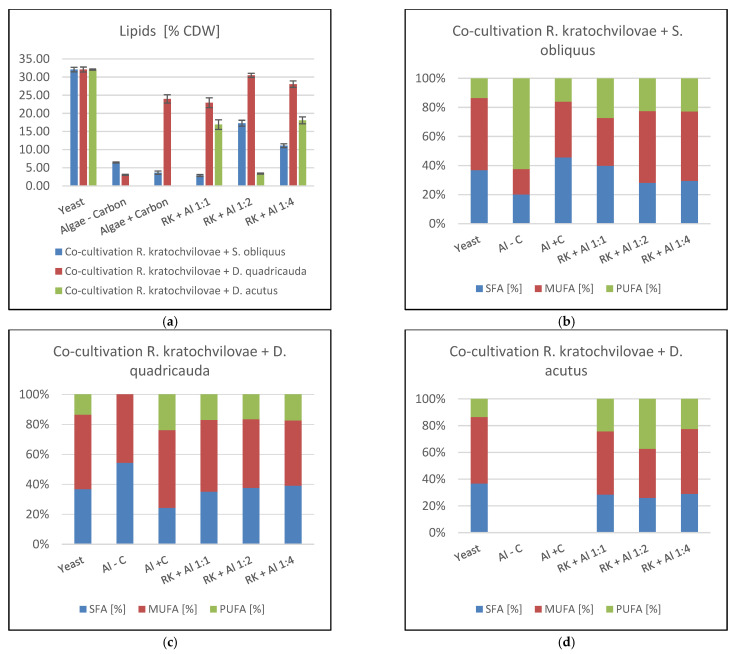
*R. kratochvilovae* lipid production and fatty acid profile in co-cultivation experiments in aerated Pyrex flasks. (**a**)—Total lipid production in all co-cultivations [%]; (**b**)—FA profile Co-cultivation *R. kratochvilovae* + *S. obliquus*; (**c**)—FA profile Co-cultivation *R. kratochvilovae* + *D. quadricauda*; (**d**)—FA profile Co-cultivation *R. kratochvilovae* + *D. acutus*. Abbreviations: Yeast—*Rhodotorula kratochvilovae* control cultivation; Algae − C—pure microalgae cultivation on BBM media without carbon source; Algae + C—pure microalgae cultivation on BBM media with carbon source; RK + AL 1:1—co-cultivation of *Rhodotorula kratochvilovae* with microalgae with inoculation ratio 1:1; RK + AL 1:1—co-cultivation of *Rhodotorula kratochvilovae* with microalgae with inoculation ratio 1:2; RK + AL 1:1—co-cultivation of *Rhodotorula kratochvilovae* with microalgae with inoculation ratio 1:4.

The results of the GC analysis of biomass samples from co-cultivation experiments are shown in the following Figure 4. The main monitored parameters were the composition of individual fatty acid groups, the percentage of lipids in the biomass, and the total lipid production. As in the previous chapters, it is not possible to evaluate and determine the general trend. In the case of co-cultivation of the yeast *R. kratochvilovae*, the highest yield of lipids was achieved in co-cultivation with *D. quadricauda*. As in pigment production results, the culture ratio of 1:2 was the best here with a total content of 30.46 ± 0.56% lipids in the biomass and thus approached the control medium. Another effect in all co-cultivation experiments of this yeast was the production of PUFA at the expense of the SFA content. MUFA production was relatively balanced in the range of 43–50% except for co-cultivation of *S. obliquus* at a ratio of 1: 1 and *D. acutus* at a ratio of 1:2.

### 3.5. Co-Cultivation of R. kratochvilovae with Microalgae in Laboratory Bioreactor

The final phase of the experiment consisted of bioreactor co-cultivation. In these co-cultivations, a microalga was inoculated into the prepared BBM medium, which was left for 24 h, and then a yeast inoculum was added to the medium. Due to the organic carbon source’s inhibitory effect on algae, the carbon source was put into the system by a peristaltic pump with a fed-batch system. During the cultivation, biomass samples were taken regularly, which were further processed and analyzed. The best pairs were cultivated in controlled bioreactor conditions using the fed-batch mode. Glycerol was added in 24 h cycles (50:25:25), and microalgae was inoculated 24 h before yeast under inoculation ratio Y:A 1:2. Co-cultivation of *R. kratochvilovae* and three *Desmodesmus* species (*D. quadricauda*, *Q. dimorphus*, *D. acutus*) in BBM media with glycerol + urea on C/N ratio 100 was performed.

#### 3.5.1. Biomass Production during Bioreactor Co-Cultivation of *Rhodotorula kratochvilovae* and *Desmodesmus* sp.

**Figure 5:**
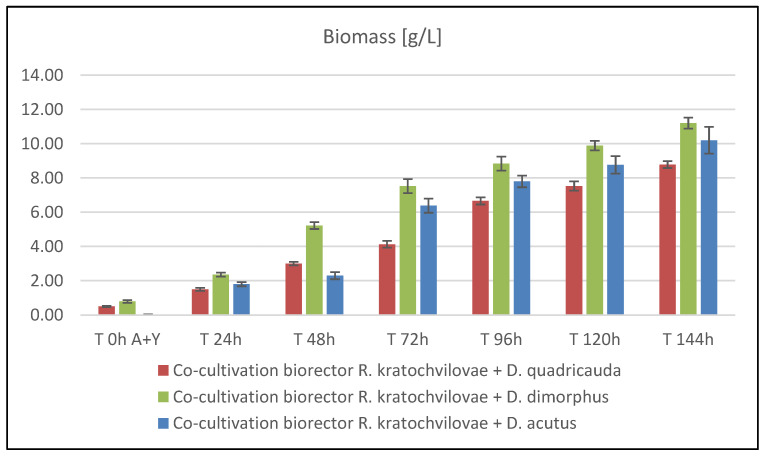
Biomass production in laboratory bioreactor co-culture of *R. kratochvilovae* and microalgae *Desmodesmus* strains.

Biomass production during co-cultivation of *R. kratochvilovae* and *D. quadricauda* in BBM media with glycerol + urea on C/N ratio 100 exhibited an exponential increase in biomass production ending at 144 h with a final production of 8.78 ± 0.20 g/L. Biomass production of co-culture of *Rhodotorula kratochvilovae* + *Desmodesmus dimorphus* C/N = 100 reached the absolute highest values of all tests performed. At the beginning of the cultivation, a rapid increase in biomass by the 66th hour of cultivation was observed, slowing down slightly but still growing. In the last sample, the production reaches 11.27 ± 0.32 g/L. It can be assumed that it would continue to grow. Co-cultivation of *Rhodotorula kratochvilovae* + *Desmodesmus acutus* C/N = 100 again provided high biomass production. Based on data measured, a linear increase in biomass throughout the experiment with a maximum (10.25 ± 0.78 g/L) in the experiment’s final phase was found. Again, we can assume that the culture would continue to grow as the experiment was extended (Figure 5).

#### 3.5.2. Cell Morphology in Co-Culture

**Figure 6:**
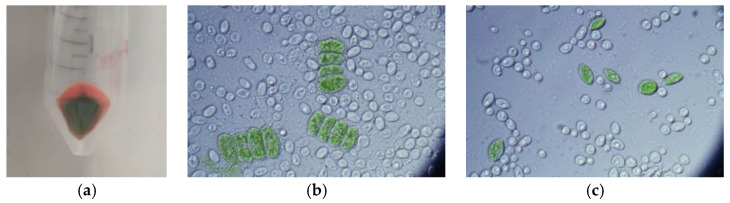
Collected biomass of co-cultivation (**a**) and microscopic images of co-culture of *Rhodotorula kratochvilovae* and *Desmodesmus quadricauda;* (**b**) *Rhodotorula kratochvilovae* and *Desmodesmus acutus* (**c**) after 96 h. Microscopic images are 100× magnified.

In Figure 6, microscopy images of co-culture of intact cells of yeasts grown together with microalgae (Figure 6b,c) in a laboratory bioreactor are documented. Biomass collected by centrifugation clearly contains yeast and microalgae portions (Figure 6a). 

#### 3.5.3. Production of Pigments, Sterols, and Quinones during Bioreactor Co-Cultivation of *Rhodotorula kratochvilovae* and *Desmodesmus* sp.

In *D. quadricauda* cells, a somewhat fluctuating trend in carotenoid production was observed over time. During the growth of the culture, three maxima can be observed in the production of carotenoids (Table 3), where the first was reached in the first stages of culture growth, at six hours, when the production reached 2.113 ± 0.024 mg/g biomass, the second maximum was reached at 48 h when production was 1.293 ± 0.019 mg/g biomass, and the third at 120 h with a 0.793 ± 0.015 mg/g biomass. Two maxima, characterized by chlorophyll production at 24 and 120 h, reached 0.703 ± 0.012 mg/g biomass at 24 h production, and at 120 h, it was 0.531 ± 0.012 mg/g biomass. Ubiquinone production after an initial decrease in the first 30 h of cultivation was followed by a high increase in production, which was the first maximum production of 5.409 ± 0.042 mg/g at 72 h. The second maximum was in the final phase, with 5.263 ± 0.078 mg/g of dry biomass production. In terms of ergosterol production, there was a linear decrease in the content of biomass dry matter. The highest production, 3.052 ± 0.038 mg/g biomass, was reached at 48 h.

During cultivation of *D. dimorphus*, carotenoid production (Table 3) reached three major maxima. The first maximum was recorded at 48 h when carotenoid production reached 1.514 ± 0.130 mg/g biomass. The second maximum was observed at 96 h of cultivation with a production value of 1.339 ± 0.099 mg/g biomass. The third and highest maximum was reached in the 140th hour of cultivation and reached the value of 2.488 ± 0.132 mg/g of biomass, which is also the highest achieved value of carotenoid production in all performed experiments. On the other hand, very low carotenoid production was observed throughout the culture, indicating that the microalgae were assimilated by yeast during the culture. The highest chlorophylls production was reached at the beginning of cultivation and reached 0.380 ± 0.024 mg/g biomass.

In *D. acutus,* the results of chromatographic analysis show a high content of pigment torularhodin in all samples. The total production of carotenoids (Table 3) is in the range of 1.5–1.9 mg/g of dry biomass, except for the sample at 24 h, where the production is very low. Production has two maxims at 46 and 120 h. The peak of ubiquinone production occurs in the final phase of the experiment at 150 h (6.088 ± 0.201 mg/g) and then at 24 h 5.939 ± 0.207 mg/g biomass. The maximum ergosterol production of 6.695 ± 0.345 mg/g of dry biomass was also measured in this sample. In other samples, we see only a decrease in ergosterol production. Chlorophyll production reached its highest value in the 20th hour of cultivation (2.108 ± 0.147 mg/g biomass), decreased from 20 to 54 h, and then increased until 120 h, reaching a significant maximum (1.165 ± 0.067 mg/g biomass). After 120 h, chlorophyll production decreased until the end of the cultivation. The combination of *Desmodesmus acutus* and *Rhodotorula kratochvilovae* achieved the highest values of chlorophyll production from all performed experiments.

#### 3.5.4. Production of Lipids and Fatty Acids during Bioreactor Co-Cultivation of *Rhodotorula kratochvilovae* and *Desmodesmus* sp.

In *D. quadricauda* cells, data show a linear decrease in the percentage of lipid content associated with increased biomass production was found. After that, however, there is a rapid linear growth in production to the final value of 29.62 ± 0.34%. The fatty acid profile during this cultivation showed relatively stable production of all three types of fatty acids. On the first day, a slight increase in PUFA production at the expense of MUFA was observed. In the final phase, a slight increase in SFA content to a total of 27.65 ± 0.08% was detected (Figure 8a). Thus, the final lipid content was 28% SFA, 46% MUFA, and 26% PUFA. The best harvesting time for the recovery of enriched biomass with all monitored components with emphasis on lipids was 144 h where the highest values of biomass growth (8.78 ± 0.20 g/L) were reached with high lipid enrichment (29.62 ± 0.34%) with the production of carotenoids (0.696 ± 0.018 mg/g biomass) and chlorophylls (0.168 ± 0.018 mg/g biomass).

As in previous experiments, high lipid production was also achieved in co-culture of *R. kratochvilovae* and *D. dimorphus*. Unlike other microalgae representatives, there is no initial decrease in the percentage of lipids, but on the contrary, increasing lipid production from the beginning was observed. During the cultivation, two maxima were reached at 90 and 144 h, the value of which exceeded 30%. After the first day of cultivation, the fatty acid profile stabilized and remained virtually unchanged until the end of the cultivation (Figure 8b). The best harvest time for achieving maximum enrichment of biomass, both with lipids and pigments, was 140 h, during which there was a significant increase in biomass (10.51 ± 0.39 g/L) and at the same time significant production of lipids (30.51%) and carotenoids (2.488 ± 0.132 mg/g of dry biomass) was measured.

**Figure 7:**
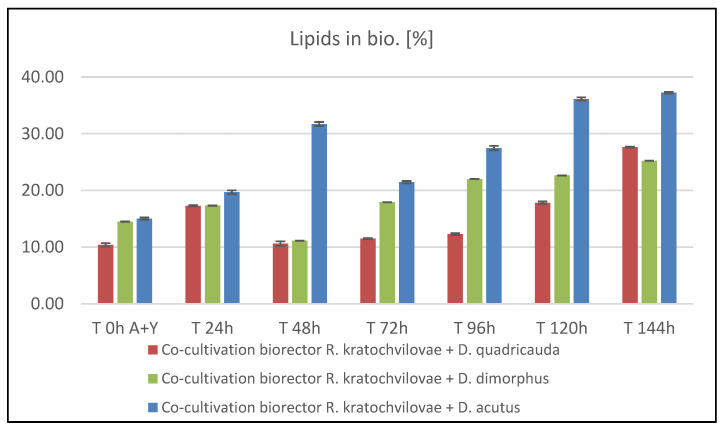
Time course of total lipid production in co-cultures of *Rhodotorula kratochvilovae* and *Desmodesmus* sp.

**Figure 8:**
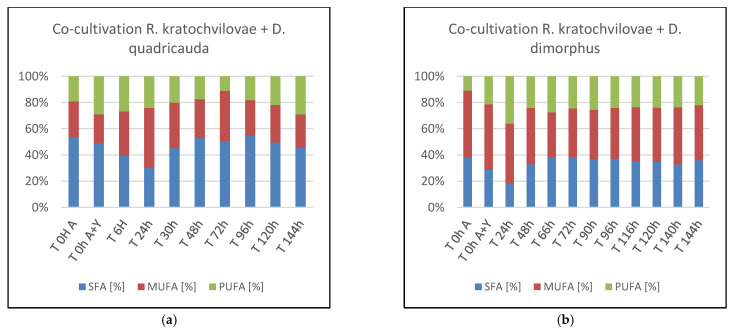

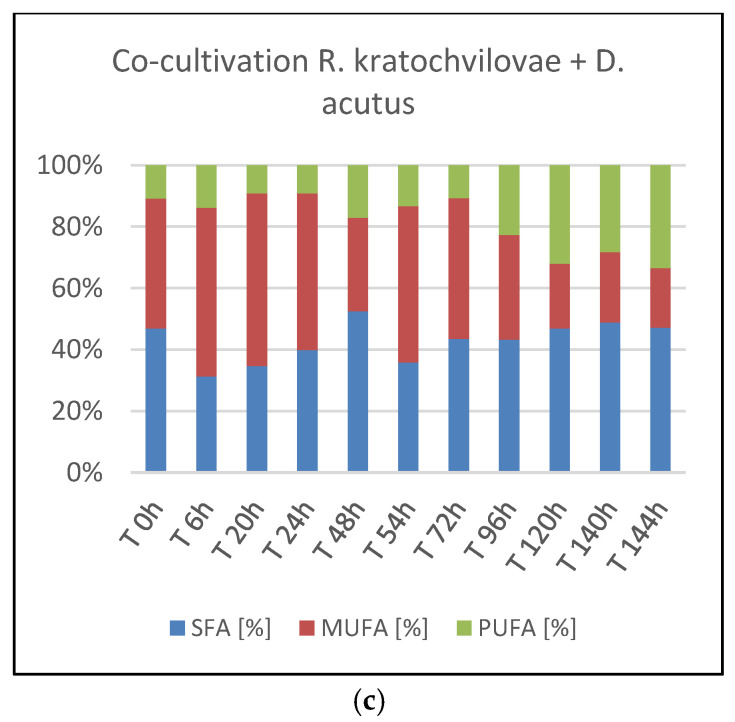
Fatty acid distribution in co-cultures of *R. kratochvilovae* and *Desmodesmus* sp. Microalgae. (**a**)—Co-cultivation *Rhodotorula kratochvilovae* with *Desmodesmus quadricauda*; (**b**)—Co-cultivation *Rhodotorula kratochvilovae* with *Desmodesmus dimorphus*; (**c**)—Co-cultivation *Rhodotorula kratochvilovae* with *Desmodesmus acutus*.

Linear biomass growth of *D. acutus* was followed by the production of lipids (Figure 7). In the first three days of cultivation, some fluctuations were observed, followed by an intensive production of lipids to a final value of 31.71 ± 0.35% in the last sample. Similar to previous cultivations, data showed a slight, almost linear decrease in PUFA content in favor of SFA. In the final stages of culture, more MUFA with SFA were accumulated in the cells, rather than PUFA (Figure 8c). The ideal harvest time to obtain biomass enriched to the maximum with pigments, and at the same time lipids, is the 120th hour when the highest production of carotenoids (1.897 ± 0.105 mg/g biomass) and chlorophyll (1.165 mg/g biomass) was achieved simultaneously with a significant biomass production (8.76 ± 0.51 g/L) and total lipid content of 30.59 ± 0.68%.

## 4. Discussion

Due to their versatile metabolic system, heterotrophic oleaginous red yeasts are capable of performing simultaneous co-production of lipids, β-glucans [20], carotenoid pigments [21], and proteins and lipids [22]. Furthermore, autotrophic microalgae are capable of performing simultaneous co-production of pigments, PUFA, β-glucans, and other bio-polymers [16]. Thus, red yeast biomass as well as microalgae biomass could be used as a high-value multifunctional biomass directly in feed and food nutrition, pharmacy, and medicine. Alternatively, the biomass could be separated and utilized in different markets that seek certain specific functionalities [11]. 

The yeast strain *R. kratochvilovae* CCY 20-2-26 [22] was selected to co-cultivation experiments based on previous screening experiments [19,23]. To set optimum conditions for bioreactor co-cultivation experiments, several experimental phases were planned. First, basic cultivations with different carbon and nitrogen sources were performed to compare yeast growth in the simple mineral medium and BBM medium used for microalgae cultures. Two sets of 4-day cultivations were followed by an experiment with 10-day cultures on BBM medium in Erlenmeyer flasks to compare the growth of yeasts with the microalgae in the subsequent phase of the cultivation. Different C (glucose, glycerol) and N sources (urea, yeast autolysate, (NH_4_)_2_SO_4_) and a mixotrophy of selected strains of microalgae were tested as well. Small-scale co-cultivation experiments in Erlenmeyer flasks were followed by co-cultivation of the most compatible strains in aerated round flasks under illumination. The most promising pairs were then co-cultivated under controlled conditions in a 3L bioreactor.

From the overall comparison, as the most suitable yeast strain in terms of biomass production, *R. kratochvilovae* CCY 20-2-26 was evaluated. In general, this yeast was the only one that managed to get over the limit of 10 g/L biomass in Erlenmeyer flasks on the BBM co-culture medium with urea. From the results, it is impossible to say unambiguously which source of carbon or nitrogen is the best. Nevertheless, ammonium sulphate appears to be unsuitable due to lowering the pH value while releasing a sulphate group into the medium followed by a gradual inhibition of growth until it stops [24]. Oppositely, yeast autolysate proved to be a suitable N source, and the results show high biomass production. However, one of the disadvantages is the high cost, which can drastically increase the whole process’ cost.

Regarding C sources, glycerol media were more suitable for carotenoid production than glucose. The carotenoid profile in all cultures of *R. kratochvilovae* was similar; the largest proportion here is β-carotene, which mostly makes up at least 30–35% of the carotenoid content. Together with beta-carotene, lycopene and torularhodin production was measured in all samples. High production of ergosterol (6.024 ± 0.098 mg/g CDW) was achieved on both media containing ammonium sulphate. Ubiquinone was identified in all samples, where the best yield was reached in a medium composed of glycerol and ammonium sulphate.

During cultivation on mineral medium, a relatively stable concentration of lipids (15% CDW) was found, and slightly higher production was measured in medium with glycerol and urea. The highest SFA content was also measured in this medium, while high PUFA production was measured in the glycerol and yeast autolysate media. Long-term cultivation on BBM media harmed lipid production in all samples to less than 10%. However, the BBM media are more suitable for PUFA production. In most cases, however, the increased PUFA production was at the expense of MUFA production, which is very low, and almost no MUFA was measured in a sample of BBM media with glycerol and yeast autolysate.

Cultivation on mineral BBM medium with a combination of carbon and nitrogen sources was crucial for further developing the experiment. A significant decrease of carotenoid and ergosterol production in BBM medium was found, compared to yeast mineral medium. As a more suitable carbon source in the BBM medium, glycerol was found. The highest production of the monitored metabolites was achieved in combination with urea, followed by the medium with ammonium sulphate and both media with yeast autolysate. Significant production of ergosterol and CoQ in medium with glycerol and urea can be noticed compared to other media. 

However, it should be noted that there are some differences between the media that lead to different productions, such as C/N ratio, pH, microelements, etc. The C/N ratio of standard BBM media is higher than in yeast mineral media. The optimal pH for yeast growth in mineral medium is around pH = 5.6. On the contrary, BBM medium has a pH of 7.0–7.1 and is therefore outside the optimal pH value that yeast prefers. Of the essential macro biogenic elements, mainly P, S, and Mg were not optimized, which can also affect the growth and production properties. Thus, the comparison of productions between both mineral and BBM media is only indicative. However, the basic media optimization took place in the final phase of cultivation in bioreactors after determining optimal combinations of strains for co-cultivation.

Microalgae biomass on the organic substrate was very low, and the strains we selected could not assimilate glucose and glycerol from the medium. In the second series, the microalgae were cultured under light for ten days. The aim was to verify whether microalgae are capable of photosynthesis in the presence of organic carbon sources. Both glucose and glycerol slightly inhibited the growth of microalgae and in a long-term experiment, the culture growth was stopped or killed. Based on these results, the co-cultivation procedure should be changed. 

Further, the microalgae were inoculated first and then the yeast and carbon source. Urea was chosen as a compromise N source. In cultures with yeast, excellent productions were achieved, and even experiments with microalgae showed compatibility. The advantage of urea is that it does not significantly affect the pH of the culture medium. After removal of the amine groups, CO_2_ is released into the media, which serves the phototrophs as a carbon source. Yeast autolysate appeared to be a suitable nitrogen source for yeast but negatively affected microalgae. In the case of microalgae, ammonium sulphate did not work at all because of pH decrease. 

Next, five different microalgae genera were tested in co-culture with *R. kratochvilovae*. Cultivation was performed in Erlenmeyer flasks in standard BBM media with glycerol and urea. The largest biomass production was achieved in the co-cultivation of yeast with *D. quadricauda.* Moreover, carotenoid and ubiquinone production increased in co-cultivations with *D. quadricauda, D. acutus*, and *S. nidulans*, respectively. Regarding algal metabolites, deficient production of chlorophylls was found. Algae probably served as a source of nutrients for the yeast in the later experiment stages, and for this reason, the vast majority of biomass was made up of yeast biomass. Generally, co-cultivation positively affects the monitored metabolites and biomass’s total production. Oppositely, in co-cultures, mostly lower production of lipids with low PUFA content was achieved. Nevertheless, optimization experiments with glycerol as a C-source led to relatively successful co-cultivations of *R. kratochvilovae* CCY 20-2-26 with all tested algae.

Based on previous experiments, co-cultivations of selected pairs were performed in aerated 1L Pyrex flasks. In each culture, yeasts were cultivated separately on BBM media with carbon sources, microalgae on BBM medium with and without a carbon source, and then co-cultivated with yeasts in three different inoculation ratios of yeast: microalgae. Previously, the yeast growth was always significantly larger than the microalgae and, thus, the yeast inoculation ratio was reduced in these experiments. The addition of a microalgae bottle to BBM medium without a carbon source was chosen as a second control to compare the effect of the carbon source on the growth of the microalgae and further study the effect of bacterial contamination on the microalgae if any. This phase of the experiment’s basic goals was to test the effect of air aeration on the production of growth and biomass of yeasts and microalgae in co-cultures and separate cultures to study the effect of different inoculation rates of yeast and microalgae and production of metabolites and lipids. 

The most successful experiment in aerated flasks was co-cultivation of *R. kratochvilovae* with *D. quadricauda*, where a higher biomass production was achieved in all co-cultivation conditions compared to the control medium. All co-cultivations exhibit higher accumulation of total carotenoid production with a different carotenoid profile—reduced β-carotene and torulene and formation of other pigments. At the same time, we see a low production of chlorophylls. Co-cultivation positively affected ubiquinone and ergosterol production. In general, co-cultivations were more productive in all proportions when compared with sole yeast culture, and the largest profits were achieved at a ratio of 1:2. In addition, cultivation of the microalgae *Desmodesmus acutus*, even in repeated experiments, led to death of pure algal culture, while in co-culture, the microalgae survived. In classical BBM medium without a carbon source, the second culture had an inhibitory effect on glucose. This phenomenon was probably due to the rapid depletion of the medium’s carbon source and a reduction in glucose’s inhibitory effect. Increased lipid and PUFA production was found in co-cultures, which overcame the low production of the sole yeast. 

The final phase of the experiment consisted of bioreactor co-cultivation. The most promising pairs—*Rhodotorula kratochvilovae* CCY 20-2-26 and three *Desmodesmus* strains (*D. quadricauda* CCALA 463, *D. dimorphus* CCALA 443*, D. acutus* CCALA 439)—were co-cultivated under controlled conditions in a 3L bioreactor in BBM media with glycerol + urea on C/N ratio 100. Previously, we have found that production of total lipids increased several times in red yeast strains at higher C/N. Oppositely, production of carotenoids, ubiquinone, and ergosterol dramatically decreased several times with an increased C/N ratio. Thus, carotenoids are produced by red yeasts, oppositely to lipids, dependent on the C/N ratio. This could be caused by the competition in the availability of acetyl-CoA as a substrate both for lipids (fatty acids) and isoprenoids (carotenoids) [8].

Due to the fact that the C/N ratio is a well-known parameter influencing yeast cellular metabolism and the production of different metabolites as well as the biomass yield [21], we tested two C/N ratios (25 and 100) in glucose and glycerol media on growth and metabolic activity of tested co-culture. The data obtained (data not shown) confirmed the previous experiment’s assumption that reducing the amount of carbon in the medium will increase the production of carotenoids and ubiquinone, while lipid production declined. Because of the high interest predominantly in the production of lipids and PUFA, co-cultivations of *Rhodotorula kratochvilovae* and *Desmodesmus* sp. were performed at C/N 100, where several times higher lipids were produced. In all co-cultures, linear biomass growth was found, where the biomass yield produced was in the range of 8.78 and 11.12 g/L. Lipid production was 29.62–31.61%. The FA profile was quite stable with the UFA portion at about 80%, while SFA gradually increased at the end of cultivation to 37%. Carotenoids slowly declined to around 2 mg/g CDW. Torularhodin was the major pigment. Ubiquinone production reached the maximum at 72 h (around 6 mg/g), while ergosterol did the same at 24 hours (6.69 ± 0.345 mg/g in co-cultivation with *D. acutus*). Chlorophyll production was very low.

Based on measured data, it can be concluded that co-cultivation of carotenogenic yeasts and microalgae has high potential for use in biotechnology. Pilot experiments showed that some strains are able to coexist and grow in one medium. Subsequent optimization of the culture conditions can significantly increase the production of the desired metabolites. The most successful experiments were achieved in the co-cultivation of the yeasts *Rhodotorula* with representatives of the genera *Desmodesmus* and Sc*enedesmus.* One of the fundamental factors of co-cultivation is the morphology of microalgae. Colonies or envelopes forming microalgae can prevent yeast ingestion in the absence of simple sources of nutrients. In the follow-up experiments, it would be appropriate to further optimize the parameters of co-cultivation and to focus more on the chemical exchange of gases and molecules between co-cultivation partners.

## 5. Conclusions

The results of the cultivations provided clear information about the possibilities of co-cultivation of yeasts and microalgae. The performed pilot cultivations also showed a positive effect of co-cultivation on the studied metabolites’ total production. They had a positive effect on the production of ubiquinone, ergosterol, and carotenoids. In lipids, we see an increased lipids production and a slightly increased content of unsaturated fatty acids compared to the classical profile in pure yeast culture. Total carotenoid production was lower due to the high C/N ratio in the media. Increased carotenoid production was achieved during trial co-cultivation with a lower C/N ratio. The lower results of some metabolites’ production compared to the mineral yeast media can be overcome by optimizing the medium’s components. Under given conditions, co-cultivations of microalgae and yeast were able to accumulate very high amounts of lipids. There is considerable potential for optimization in this area. The absolute best bioreactor results were obtained in the co-cultivation of the yeast *R. kratochvilovae* and representatives of the genera *Desmodesmus* and *Scenedesmus*. High lipid production was achieved in these co-cultivations, where the percentage of lipids in the biomass was always higher than 30%. The highest production of lipids was achieved by co-cultivation of *R. kratochvilovae* and *D. quadricauda*. In the end, it can be concluded that the goals set for this experiment have been achieved. Pilot experiments have confirmed that carotenogenic yeasts and microalgae are capable of symbiotic coexistence.

## Figures and Tables

**Table 1:** Experimental scheme of first co-cultivation phase.

Yeast Mineral Cultivation Media						
	Flask 1.	Flask 2.	Flask 3.	Flask 4.	Flask 5.	Flask 6.
Carbon source	Glucose	Glucose	Glucose	Glycerol	Glycerol	Glycerol
Nitrogen source	Urea	(NH_4_)_2_SO_4_	Yeast autolysate	Urea	(NH_4_)_2_SO_4_	Yeast autolysate
BBM mineral media						
Carbon source	Glucose	Glucose	Glucose	Glycerol	Glycerol	Glycerol
Nitrogen source	Urea	(NH_4_)_2_SO_4_	Yeast autolysate	Urea	(NH_4_)_2_SO_4_	Yeast autolysate

**Table 2:** Production of pigments and sterols by *R. kratochvilovae* co-cultivated with representatives of the genera *Desmodesmus* and *Scenedesmus* in aerated pyrex flasks (mg/g CDW).

Part A: *Rhodotorula kratochvilovae* Co-Cultivated with *Desmodesmus quadricauda*								
Strain	Betacarotene	Lutein	Torulene	Total Carotenoids	Chlorophyll A	Chlorophyll B	Co Q	Ergosterol
*R.kratochvilovae*	0.224 ± 0.015	0	0.706 ± 0.096	1.399 ± 0.156	0	0	2.400 ± 0.186	5.439 ± 0.239
DQ − C	1.256 ± 0.135	0.271 ± 0.051	0	4.403 ± 0.236	1.255 ± 0.083	1.068 ± 0.086	3.333 ± 0.198	0
DQ + C	0.780 ± 0.096	0.159 ± 0.023	0	5.491 ± 0.267	0.105 ± 0.016	0.051 ± 0.008	1.888 ± 0.156	0
RK+DQ 1:1	0.406 ± 0.056	0.001 ± 0.001	1.367 ± 0.064	3.976 ± 0.196	0.234 ± 0.013	0	6.035 ± 0.213	5.370 ± 0.256
RK+DQ 1:2	2.231 ± 0.136	0.136 ± 0.026	0.656 ± 0.081	11.221 ± 0.362	0.097 ± 0.009	0.038 ± 0.004	3.325 ± 0.106	2.954 ± 0.186
RK+DQ 1:4	0.592 ± 0.089	0.041 ± 0.006	1.332 ± 0.046	3.837 ± 0.185	0.167 ± 0.026	0.187 ± 0.021	3.452 ± 0.093	3.758 ± 0.125
**Part B: *Rhodotorula kratochvilovae* Co-Cultivated with *Desmodesmus acutus***								
**Strain**	**Betacarotene**	**Lutein**	**Torularhodin**	**Total Carotenoids**	**Chlorophyll A**	**Chlorophyll B**	**Co Q**	**Ergosterol**
RK	0.022 ± 0.008	0	0.013 ± 0.003	0.162 ± 0.033	0	0	0.240 ± 0.018	0.544 ± 0.061
RK + DA 1:1	0.401 ± 0.064	0.026 ± 0.008	0.617 ± 0.053	2.969 ± 0.126	0.518 ± 0.023	0.303 ± 0.032	3.390 ± 0.136	4.634 ± 0.139
RK + DA 1:2	1.241 ± 0.093	0.103 ± 0.013	0.210 ± 0.039	3.790 ± 0.157	3.176 ± 0.094	1.010 ± 0.091	4.565 ± 0.183	2.638 ± 0.094
RK + DA 1:4	0.562 ± 0.066	0.057 ± 0.010	0.625 ± 0.065	3.339 ± 0.113	1.106 ± 0.106	0.623 ± 0.076	1.563 ± 0.121	4.196 ± 0.144
**Part C: *Rhodotorula kratochvilovae* Co-Cultivated with *Scenedesmus obliquus***								
	**Betacarotene**	**Lutein**	**Torularhodin**	**Total Carotenoids**	**Chlorophyll A**	**Chlorophyll B**	**Coenzyme Q**	**Ergosterol**
RK	0.224 ± 0.036	0	0.134 ± 0.023	1.621 ± 0.072	0	0	2.400 ± 0.112	5.439 ± 0.239
SO − C	3.344 ± 0.103	5.090 ± 0.232	0	13.063 ± 0.361	13.277 ± 0.226	7.589 ± 0.245	0.263 ± 0.013	0
SO + C	1.811 ± 0.096	0.114 ± 0.019	0	3.293 ± 0.134	0.599 ± 0.049	0.292 ± 0.054	0.841 ± 0.036	0
RK + SO 1:1	4.660 ± 0.236	0.115 ± 0.010	0.175 ± 0.023	7.458 ± 0.176	2.058 ± 0.131	0.850 ± 0.068	4.733 ± 0.099	2.129 ± 0.083
RK + SO 1:2	0.571 ± 0.083	0.057 ± 0.006	0.496 ± 0.061	2.756 ± 0.153	0.305 ± 0.026	0.174 ± 0.012	3.309 ± 0.135	5.518 ± 0.222
RK + SO 1:4	0.836 ± 0.069	0.099 ± 0.007	0.468 ± 0.043	3.409 ± 0.138	0.679 ± 0.044	0.293 ± 0.033	3.448 ± 0.112	4.967 ± 0.164

Note: Concentrations of metabolites are listed in mg/g biomass. Abbreviations: RK—*Rhodotorula kratochvilovae*; SO—*Scenedesmus obliquus*, DA—*Desmodesmus acutus*; DQ—*Desmodesmus quadricauda*; −C—cultivation without carbon source in media, +C—cultivation with carbon source; 1:1 1:2 1:4—inoculation ratios.

**Table 3:** Bioreactor co-cultivation *Rhodotorula kratochvilovae* + *Desmodesmus* sp.—production of pigments and sterols during the growth (mg/g CDW).

*Rhodotorulakratochvilovae* + *Desmodesmus quadricauda*							
Time	Lutein	Torularhodin	Betacarotene	Total Carotenoids	Total Chlorophylls	Ergosterol	Ubiquinone
T 6 h	0.097 ± 0.008	1.617 ± 0.003	0.216 ± 0.007	2.113 ± 0.024	0.425 ± 0.058	5.621 ± 0.036	4.445 ± 0.038
T 24 h	0.128 ± 0.012	0.988 ± 0.005	0.225 ± 0.010	1.484 ± 0.018	0.703 ± 0.012	2.683 ± 0.042	2.058 ± 0.026
T 30 h	0.061 ± 0.009	0.645 ± 0.008	0.131 ± 0.008	0.960 ± 0.020	0.479 ± 0.017	1.465 ± 0.040	1.253 ± 0.015
T 48 h	0.061 ± 0.011	0.948 ± 0.010	0.163 ± 0.017	1.293 ± 0.019	0.388 ± 0.009	3.052 ± 0.038	4.785 ± 0.035
T 72 h	0.048 ± 0.006	0.670 ± 0.004	0.145 ± 0.006	1.050 ± 0.021	0.327 ± 0.006	2.600 ± 0.028	5.409 ± 0.042
T 96 h	0.007 ± 0.002	0.129 ± 0.013	0.032 ± 0.016	0.291 ± 0.007	0.101 ± 0.010	0.779 ± 0.013	2.650 ± 0.032
T 120 h	0.039 ± 0.003	0.373 ± 0.017	0.150 ± 0.009	0.793 ± 0.015	0.531 ± 0.012	2.201 ± 0.017	4.146 ± 0.061
T 144 h	0.015 ± 0.007	0.119 ± 0.007	0.045 ± 0.006	0.696 ± 0.018	0.168 ± 0.018	1.936 ± 0.010	5.263 ± 0.078
***Rhodotorula kratochvilovae + Desmodesmus dimorphus***							
**Time**	**Lutein**	**Torularhodin**	**Betacarotene**	**Total Carotenoids**	**Total Chlorophylls**	**Ergosterol**	**Ubiquinone**
T 0 h A+Y	0.027 ± 0.003	0.120 ± 0.003	0.129 ± 0.005	0.307 ± 0.011	0.380 ± 0.024	2.343 ± 0.102	5.417 ± 0.187
T 24 h	0.023 ± 0.004	0.189 ± 0.007	0.135 ± 0.008	0.539 ± 0.065	0.225 ± 0.019	1.106 ± 0.099	3.784 ± 0.164
T 48 h	0.022 ± 0.002	1.180 ± 0.045	0.119 ± 0.021	1.514 ± 0.130	0.203 ± 0.034	3.315 ± 0.085	3.622 ± 0.0155
T 66 h	0.022 ± 0.003	0.585 ± 0.065	0.054 ± 0.010	0.762 ± 0.087	0.215 ± 0.015	1.580 ± 0.107	2.201 ± 0.087
T 72 h	0.024 ± 0.007	0.720 ± 0.047	0.222 ± 0.33	1.115 ± 0.106	0.244 ± 0.065	2.420 ± 0.074	4.861 ± 0.174
T 90 h	0.031 ± 0.006	0.500 ± 0.035	0.112 ± 0.012	1.061 ± 0.067	0.202 ± 0.042	2.368 ± 0.102	2.501 ± 0.080
T 96 h	0.051 ± 0.010	0.984 ± 0.048	0.103 ± 0.008	1.339 ± 0.099	0.307 ± 0.018	2.609 ± 0.140	2.123 ± 0.103
T 116 h	0.080 ± 0.012	0.542 ± 0.025	0.255 ± 0.041	0.998 ± 0.084	0.197 ± 0.008	2.498 ± 0.109	2.342 ± 0.090
T 120 h	0.091 ± 0.013	0.760 ± 0.033	0.278 ± 0.034	1.286 ± 0.108	0.182 ± 0.011	3.003 ± 0.132	4.364 ± 0.105
T 140 h	0.103 ± 0.015	1.881 ± 0.098	0.191 ± 0.015	2.488 ± 0.132	0.172 ± 0.023	4.787 ± 0.140	4.568 ± 0.166
T 144 h	0.120 ± 0.020	1.111 ± 0.102	0.285 ± 0.024	1.785 ± 0.102	0.161 ± 0.020	3.538 ± 0.108	4.587 ± 0.129
***Rhodotorula kratochvilovae + Desmodesmus acutus***							
**Time**	**Lutein**	**Torularhodin**	**Betacarotene**	**Total Carotenoids**	**Total Chlorophylls**	**Ergosterol**	**Ubiquinone**
T 20 h	0.066 ± 0.010	1.165 ± 0.038	0.183 ± 0.008	1.977 ± 0.102	2.108 ± 0.147	3.863 ± 0.201	2.102 ± 0.102
T 24 h	0.109 ± 0.011	0.170 ± 0.017	0.059 ± 0.017	0.831 ± 0.064	1.516 ± 0.123	6.695 ± 0.345	5.939 ± 0.207
T 46 h	0.037 ± 0.006	1.420 ± 0.078	0.204 ± 0.013	1.856 ± 0.111	1.471 ± 0.098	3.689 ± 0.187	3.174 ± 0.190
T 54 h	0.053 ± 0.007	0.970 ± 0.065	0.113 ± 0.007	1.474 ± 0.107	0.931 ± 0.071	4.118 ± 0.158	4.385 ± 0.183
T 72 h	0.094 ± 0.018	1.085 ± 0.098	0.140 ± 0.021	1.832 ± 0.098	1.091 ± 0.103	4.895 ± 0.201	3.462 ± 0.146
T 96 h	0.030 ± 0.006	0.874 ± 0.074	0.097 ± 0.007	1.149 ± 0.079	0.445 ± 0.021	2.956 ± 0.162	3.527 ± 0.078
T 120 h	0.090 ± 0.007	1.190 ± 0.102	0.267 ± 0.023	1.897 ± 0.105	1.165 ± 0.067	2.571 ± 0.089	4.568 ± 0.128
T 140 h	0.031 ± 0.013	0.922 ± 0.086	0.103 ± 0.012	1.212 ± 0.67	0.469 ± 0.035	3.119 ± 0.134	3.722 ± 0.136
T 150 h	0.044 ± 0.008	1.093 ± 0.074	0.118 ± 0.023	1.521 ± 0.101	0.420 ± 0.045	3.083 ± 0.156	6.088 ± 0.201

## Supplementary Materials

The following are available online at: https://www.mdpi.com/article/10.3390/microorganisms9061160/s1, Figure S1: *Rhodotorula kratochvilovae* growth on different media types: lipid production and fatty acid profile GC analysis; Figure S2: *R. kratochvilovae* lipid production in small-scale co-cultivation experiments; Figure S3: *R. kratochvilovae* fatty acid profile in small-scale co-cultivation experiments; Table S1: 2nd Phase of co-cultivation experimental scheme; Table S2: Experimental scheme of 4th phase of co-cultivation; Table S3: Bioreactor process values during co-cultivation; Table S4: HPLC gradient analysis; Table S5: Temperature programme of GC analysis; Table S6: HPLC analysis of *R. kratochvilovae*: 4-day cultivation on standard yeast media (Part A) and 10-day cul-tivation on BBM media (Part B). Concentrations of metabolites are listed in mg/g of dry biomass; Table S7: *R. kratochvilovae* small-scale 10-day co-cultivation on BBM media (glucose + urea).
