# Supplementary material for: Bioreactor Co-Cultivation of High Lipid and Carotenoid Producing Yeast Rhodotorula kratochvilovae and Several Microalgae under Stress
Source: Microorganisms. 2021 May 28;9(6):1160. doi: 10.3390/microorganisms9061160 (PMC8228999; doi:10.3390/microorganisms9061160)
Supplement: Supplementary file 1 [file microorganisms-09-01160-s001.zip › microorganisms-1224739-supplementary.pdf]

microorganisms

*Suplementary material*

# Bioreactor Co-Cultivation of High Lipid and Carotenoid Producing Yeast *Rhodotorula kratochvilovae* and Several Microalgae under Stress

**Martin Szotkowski *, Jiří Holub, Samuel Šimanský, Klára Hubačová, Pavlína Sikorová, Veronika Mariničová, Andrea Němcová and Ivana Márová**

Faculty of Chemistry, Brno University of Technology, 612 00 Brno, Czech Republic; xcholubj@vutbr.cz (J.H.); xcsimansky@vutbr.cz (S.Š.); xchuba-cova@vutbr.cz (K.H.); xcsikorovap@vutbr.cz (P.S.);
xcmarincova@fch.vut.cz (V.M.); andrea.nemcova@fch.vut.cz (A.N.); marova@fch.vut.cz (I.M.)
* Correspondence: xcszotkowski@fch.vut.cz; Tel.: +420-775-288-439

**Contents:**

**Figure S1** *Rhodotorula kratochvilovae* growth on different media types: lipid production and fatty acid profile GC analysis
**Figure S2** *R. kratochvilovae* lipid production in small-scale co-cultivation experiments
**Figure S3** *R. kratochvilovae* fatty acid profile in small-scale co-cultivation experiments

**Table S1** 2nd Phase of co-cultivation experimental scheme
**Table S2** Experimental scheme of $4^{th}$ phase of co-cultivation
**Table S3** Bioreactor process values during co-cultivation
**Table S4** HPLC gradient analysis
**Table S5** Temperature programme of GC analysis
**Table S6** HPLC analysis of *R. kratochvilovae*: 4-day cultivation on standard yeast media (Part A) and 10-day cultivation on BBM media (Part B). Concentrations of metabolites are listed in mg/g of dry biomass.
**Table S7** *R. kratochvilovae* small-scale 10-day co-cultivation on BBM media (glucose + urea).

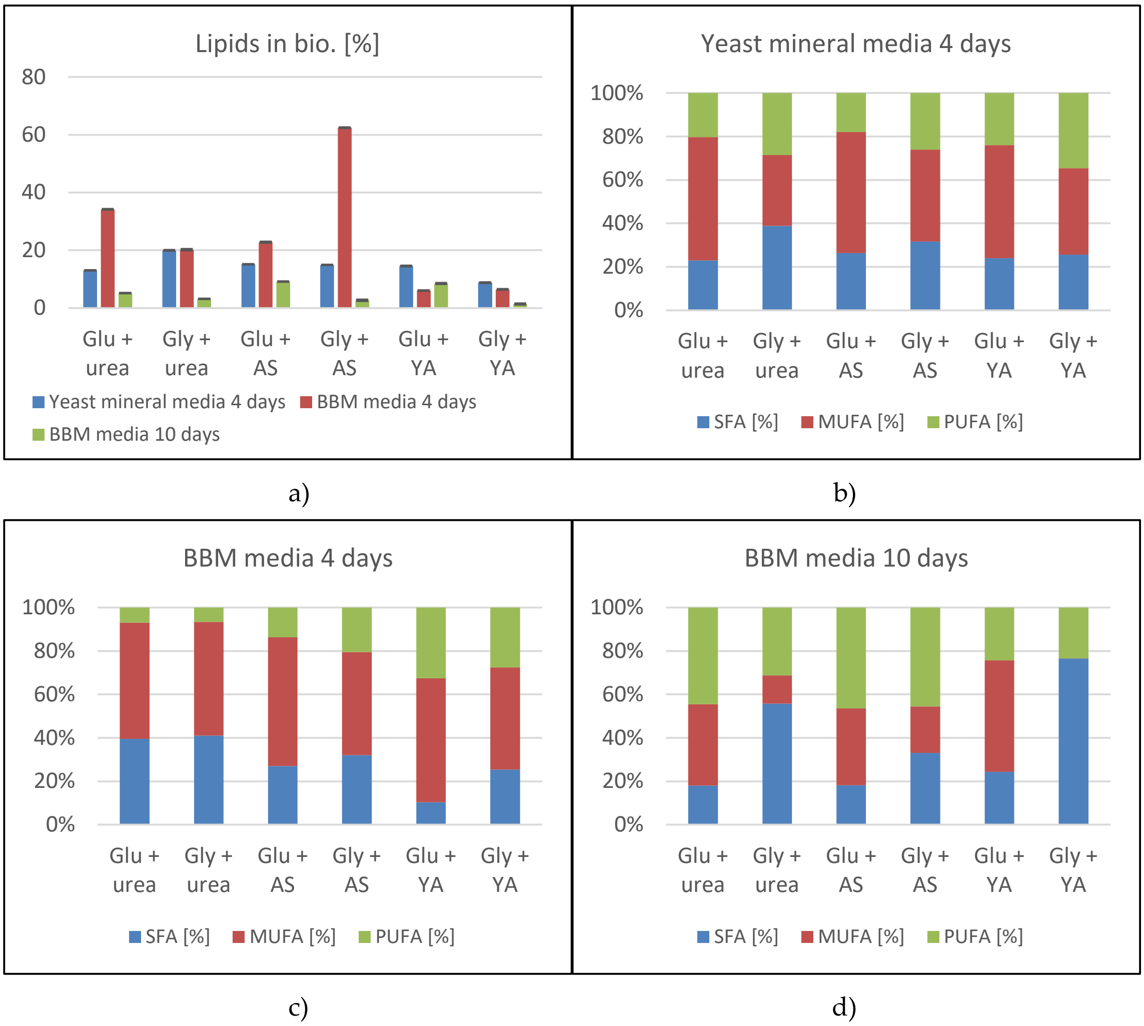


Figure S1. *Rhodotorula kratochvilovae* growth on different media types: lipid production and fatty acid profile GC analysis

a) - Total lipid production [%] b) – FA profile on yeast mineral media 4-day cultivation; c) – FA profile on BBM media 4 days cultivation; d) – FA profile on BBM media 10 days cultivation.

Abbreviations: Glu – Glucose, Gly – Glycerol, AS – Ammonium sulphate, YA – Yeast autolysate

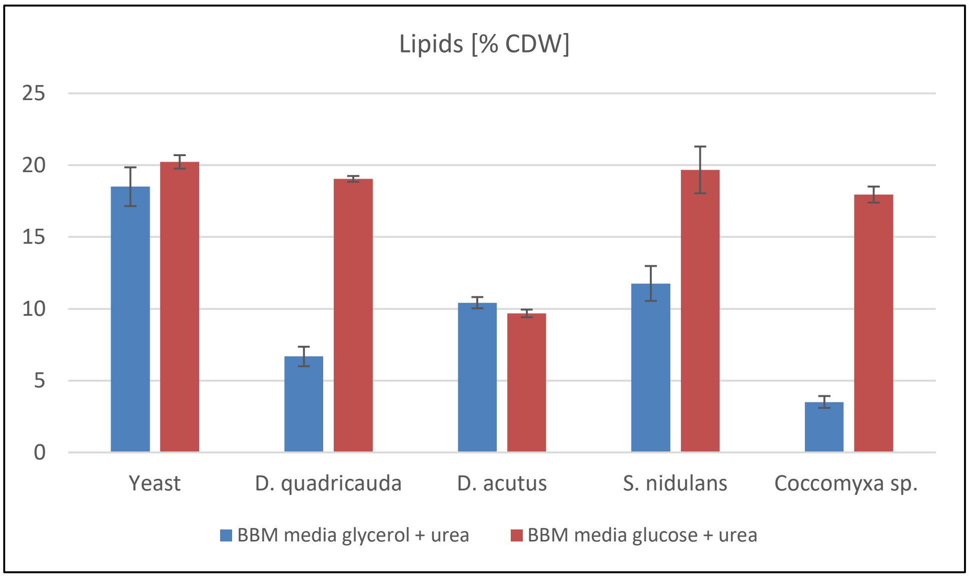

Figure S2. *R. kratochvilovae* lipid production in small-scale co-cultivation experiments

Abbreviations: Yeast – *Rhodotorula kratochvilovae* control cultivation; D. quadricauda – Co-cultivation with *Desmodesmus quadricauda*; D. acutus – Co-cultivation with *Desmodesmus acutus*; S. nidulans– Co-cultivation with *Synechococcus nidulans*; Coccomyxa sp. – Co-cultivation with *Coccomyxa sp.*

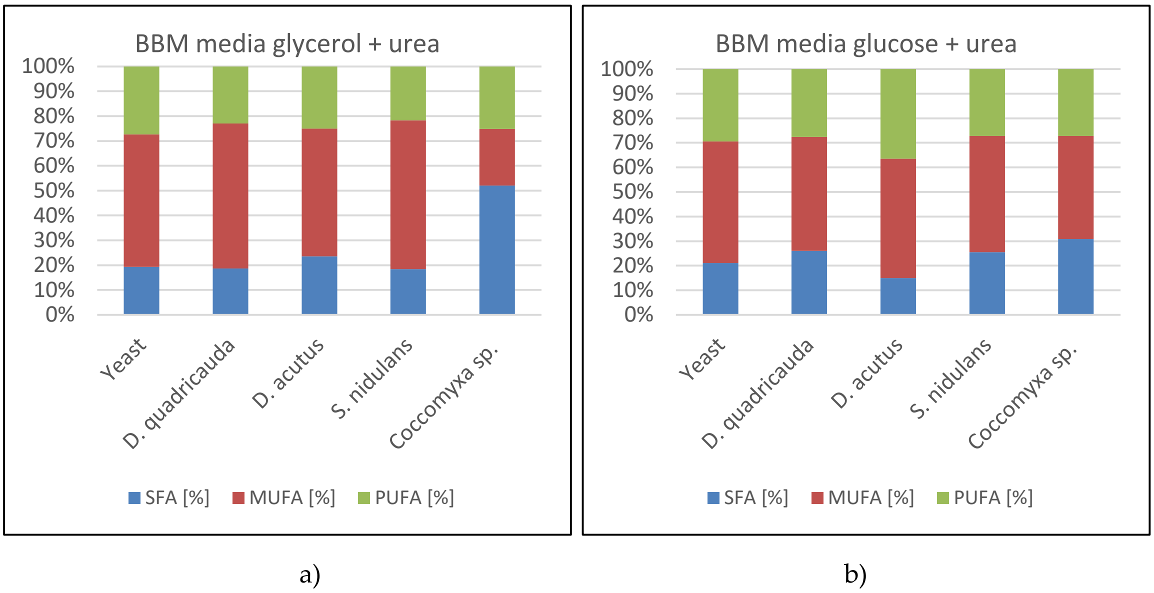


Figure S3. *R. kratochvilovae* fatty acid profile in small-scale co-cultivation experiments

a) - FA profile analysis of *Rhodotorula kratochvilovae* co-cultivation on BBM media with glycerol and urea; b) - - FA profile analysis of *Rhodotorula kratochvilovae* co-cultivation on BBM media with glucose and urea

Abbreviations: Yeast – *Rhodotorula kratochvilovae* control cultivation; D. quadricauda – Co-cultivation with *Desmodesmus quadricauda*; D. acutus – Co-cultivation with *Desmodesmus acutus*; S. nidulans– Co-cultivation with *Synechococcus nidulans*; Coccomyxa sp. – Co-cultivation with *Coccomyxa sp.*

Table S1. 2nd Phase of co-cultivation experimental scheme

| BBM mineral media | | | | | | | |
|---|---|---|---|---|---|---|---|
| Carbon source | Glucose | Glucose | Glucose | Glycerol | Glycerol | Glycerol | - |
| Nitrogen source | Urea | $(NH_4)_2SO_4$ | $NaNO_3$ | Urea | $(NH_4)_2SO_4$ | $NaNO_3$ | $NaNO_3$ |

Table S2. Experimental scheme of 4th phase of co-cultivation

| Yeast | Yeast: Algae 1:1 | Yeast: Algae 1:2 | Yeast: Algae 1:4 | Algae | Algae |
|---|---|---|---|---|---|
| BBM+ C | BBM+ C | BBM+ C | BBM+ C | BBM+ C | BBM |

Table S3. Bioreactor process values during co-cultivation

| | |
|---|---|
| Media volume | 2.0 L |
| Stirring | 300 – 800 rpm – regulated by oxygen consumption |
| pH | 6.5 |
| $pO_2$ | 30% |
| Temperature | 22 °C |

| Aeration | 2 L per minute |
|---|---|
| Illumination | 300 ol·m²·s⁻¹ of photons |

Table S4. HPLC gradient analysis

| Time [min] | Mobile phase A | Mobile phase B |
|---|---|---|
| 0.0 | 100 % | 0 % |
| 13.0 | 0 % | 100 % |
| 19.0 | 0 % | 100 % |
| 20.0 | 100 % | 0 % |
| 25.0 | 100 % | 0 % |

Table S5. Temperature programme of GC analysis

| | *Retention time (min)* | *gradient (°C·min⁻¹)* | *temperature (°C)* | *retention (min)* |
|---|---|---|---|---|
| **1** | 0.000 | start | - | - |
| **2** | 1.000 | 0.0 | 80.0 | 1.0 |
| **3** | 5.000 | 15.0 | 140.0 | 0.0 |
| **4** | 21.667 | 3.0 | 190.0 | 0.0 |
| **5** | 25.467 | 25.0 | 260.0 | 1.0 |
| **6** | 25.467 | stop | - | - |

Table S6. HPLC analysis of *R. kratochvilovae*: 4-day cultivation on standard yeast media (Part A) and 10-day cultivation on BBM media (Part B). Concentrations of metabolites are listed in mg/g of dry biomass.

Part A 4-day cultivation on yeast media

| | Betacarotene | Torulene | Torularhodin | Total carote-noids | Ergosterol | Coenzyme Q |
|---|---|---|---|---|---|---|
| Glu + urea | 2.044±0.082 | 0.161±0.065 | 0.649±0.057 | 4.929±0.067 | 3.198±0.101 | 1.764±0.103 |
| Gly + urea | 3.626±0.078 | 0.140±0.059 | 0.885±0.082 | 6.637±0.088 | 4.485±0.127 | 0.066±.0114 |
| Gly + AS | 2.649±0.071 | 0.439±0.064 | 0.688±0.091 | 7.806±0.097 | 6.024±0.098 | 0.786±0.106 |
| Glu + AS | 1.880±0.058 | 0.083±0.071 | 0.518±0.087 | 5.356±0.048 | 5.091±0.108 | 1.166±0.099 |
| Gly +YA | 1.886±0.083 | 0.114±0.058 | 0.379±0.085 | 6.366±0.071 | 3.941±0.078 | 1.262±0.120 |
| Glu +YA | 1.417±0.078 | - | 0.033±0.072 | 3.055±0.083 | 2.350±0.106 | 2.041±0.089 |

Part B 10-day cultivation on BBM media

| | Betacarotene | Torulene | Torularhodin | Total carote-noids | Ergosterol | Coenzyme Q |
|---|---|---|---|---|---|---|
| Glu + urea | 0.434±0.102 | 0.014±0.028 | 0.088±0.078 | 0.963±0.0 97 | 0.922±0.070 | 0.241±0.043 |
| Gly + urea | 0.841±0.097 | 0.161±0.034 | 0.345±0.064 | 3.011±0.142 | 4.017±0.103 | 0.937±0.081 |
| Gly + AS | 0.425±.0087 | 0.115±0.037 | 0.347±0.047 | 2.123±0.131 | 1.804±0.074 | 0.939±0.078 |
| Glu + AS | 0.175±0.039 | - | 0.041±0.032 | 0.455±0.087 | 1.624±0.099 | 0.396±0.068 |
| Gly +YA | 0.574±.0114 | - | 0.181±0.049 | 1.425±0.076 | 1.810±0.086 | 0.180±0.047 |
| Glu + YA | 0.709±0.106 | 0.066±0.047 | 0.210±0.037 | 1.809±0.088 | 2.116±0.047 | 0.347±0.065 |

Abbreviations: Glu – Glucose, Gly – Glycerol, AS – Ammonium sulphate, YA – Yeast autolysate

Table S7. *R. kratochvilovae* small-scale 10-day co-cultivation on BBM media (glucose + urea).

| Strain | Betacarotene | Lutein | Total carote-noids | chlorophyll A | Chlorophyll B | Coenzyme Q | Ergosterol |
|---|---|---|---|---|---|---|---|
| *S. nidulans* | 0.276±0.032 | 0.002±0.001 | 1.096±0.163 | 0.052±0.009 | 0.810±0.048 | 1.424±0.103 | 2.198±0.135 |
| *D. quadricauda* | 0.798±0.103 | 0.014±0.006 | 2.191±0.207 | 0.092±0.007 | 0.011±0.002 | 0.980±0.046 | 1.662±0.086 |
| *Coccomyxa sp.* | 0.209±0.025 | 0.004±0.002 | 0.645±0.103 | 0.020±0.005 | 0.018±0.006 | 0.820±0.060 | 1.444±0.204 |
| *D. acutus* | 0.850±0.097 | 0.008±0.003 | 2.133±0.182 | 0.140±0.032 | 0.043±0.018 | 2.417±0.146 | 3.067±0.148 |
| *R. kratochvilovae* | 0.702±0.121 | 0 | 1.827±0.065 | 0 | 0 | 1.248±0.097 | 1.585±0.115 |

**Note:** Concentrations of metabolites are listed in mg/g biomass.
